# Gastroblastoma mimics the embryonic mesenchyme of the foregut: a case report

**DOI:** 10.1186/s13000-023-01310-2

**Published:** 2023-02-17

**Authors:** Ryo Sugimoto, Noriyuki Uesugi, Noriyuki Yamada, Mitsumasa Osakabe, Shigeaki Baba, Naoki Yanagawa, Yuji Akiyama, Wataru Habano, Akira Sasaki, Yoshinao Oda, Tamotsu Sugai

**Affiliations:** 1 Department of Molecular Diagnostic Pathology, School of Medicine, Iwate Medical University, 2-1-1 Idaitoiri, Yahaba, Shiwa, Iwate, Japan; 2 Department of Surgery, School of Medicine, Iwate Medical University, Shiwa, Japan; 3 Division of Pharmacodynamics and Molecular Genetics, School of Pharmacy, Iwate Medical University, Shiwa, Japan; 4 Department of Anatomic Pathology, Pathological Science, Graduate School of Medical Science, Kyusyu University, Fukuoka, Japan

**Keywords:** Gastroblastoma, *MALAT1-GLI* fusion gene, PD-L1

## Abstract

**Background:**

Gastroblastoma is a rare gastric tumor composed of epithelial and spindle cell components. The characteristic *MALAT–GLI1* fusion gene has only been identified in 5 reported cases. We report the morphological characterization of gastroblastoma with the *MALAT1–GLI1* fusion gene in a young Japanese woman.

**Case presentation:**

A 29-year-old Japanese woman visited Iwate Medical University Hospital with upper abdominal pain. Computed tomography revealed a tumor in expansive lesions involving the gastric antrum. Histologically, we observed a biphasic morphology composed of epithelial and spindle cell components. The epithelial components appeared as slit-like glandular structures with tubular or rosette-like differentiation. The spindle cell components consisted of short spindle-shaped oval cells. Immunohistochemical (IHC) analysis revealed that the spindle cell component was positive for vimentin, CD10, CD56, GLI1, and HDAC2, and focally positive for PD-L1. The epithelial component was positive for CK AE1/AE3, CAM5.2, and CK7, and negative for CK20 and EMA. Both components were negative for KIT, CD34, DOG1, SMA, desmin, S100 protein, chromogranin A, synaptophysin, CDX2, and SS18-SSX. The *MALAT-GLI1* fusion gene was detected molecularly.

**Conclusions:**

We report the following new findings with this case: (i) gastric tumors mimic the gastrointestinal mesenchyme in the embryonic period; (ii) nuclear expression of PD-L1 and HDAC2 were observed in the spindle cell component of a gastroblastoma. We speculate that histone deacetylase (HDAC) inhibitors may offer a promising treatment option for gastroblastoma.

## Background

Gastroblastoma, first described by Miettinen and colleagues in 2009, is a rare gastric tumor characterized by epithelial and spindle cell components [1]. To our knowledge, 16 cases have been reported in the medical literature [1–11]. However, the characteristic *MALAT1–GLI1* fusion gene was identified in only 5 of those cases. Due to the rarity of this disease, its pathogenesis remains unknown. We report a case of gastroblastoma containing a *MALAT1–GLI1* fusion gene in a young Japanese woman.

## Case presentation

**Fig. 1:**
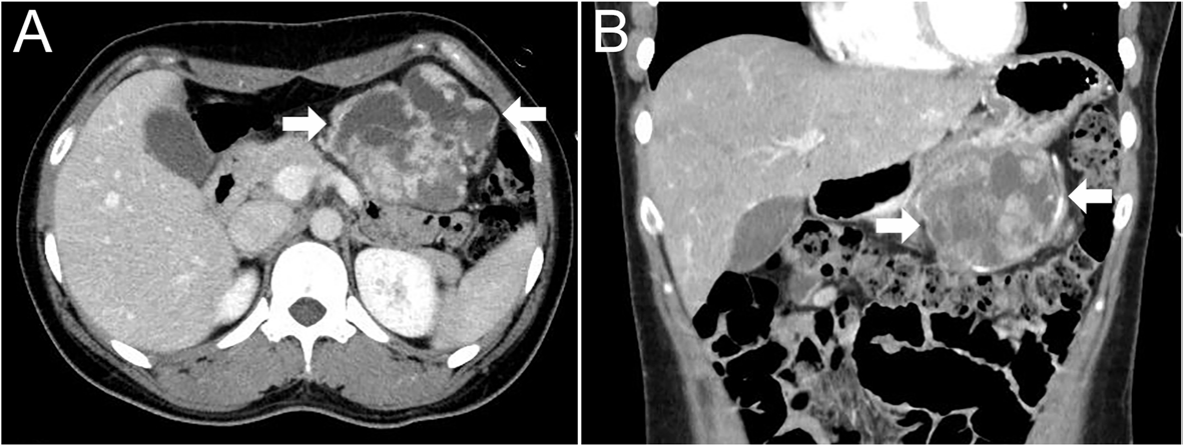
A 29-year-old Japanese woman with gastroblastoma. **A** Axial plane enhanced computed tomography (CT) image of the abdomen. The gastroblastoma appears as a well-circumscribed mass (arrows) showing a multi-cystic, heterogeneously enhanced mass in the lower part of the stomach; **B** Coronal plane enhanced CT image of the abdomen. Multi-cystic nature and heterogeneity of the gastroblastoma (arrows) arising from the bottom of the gastric wall in the upper-left quadrant

A 29-year-old Japanese woman visited Iwate Medical University Hospital with upper abdominal pain for over a week. The patient had no surgical history, drug allergies, or family history of malignancy. Her laboratory examination was unremarkable. A contrast-enhanced computed tomography (CT) scan showed a 70-mm antral expansive mass (Figs. 1A, B). A biopsy of the tumor was not performed, and she was diagnosed clinically with a gastrointestinal stromal tumor (GIST). Although a preoperative histological diagnosis was not made, a laparoscopic partial gastrectomy was performed. Eight months have passed since the surgery; however, no recurrence or metastasis has been found.

**Fig. 2:**
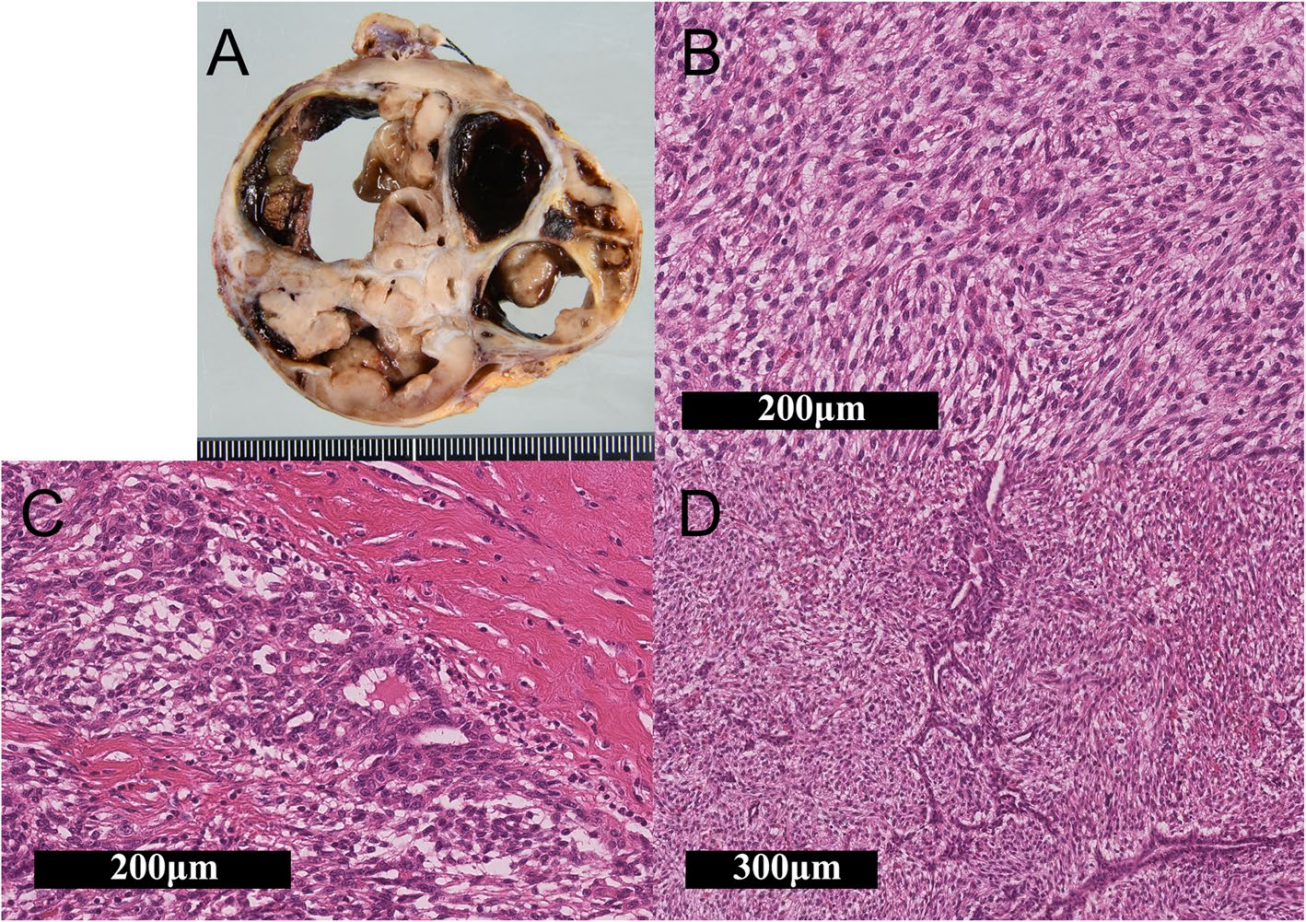
Cut surface and histology of the gastroblastoma. **A** Cut surface of the gastroblastoma. **B** Most of the tumor cells were spindle cells, which appeared oval-shaped without atypia (x200). **C** Tubular or rosette-like differentiation (x200). **D** Glandular and slit-like structure (x100)

**Fig. 3:**
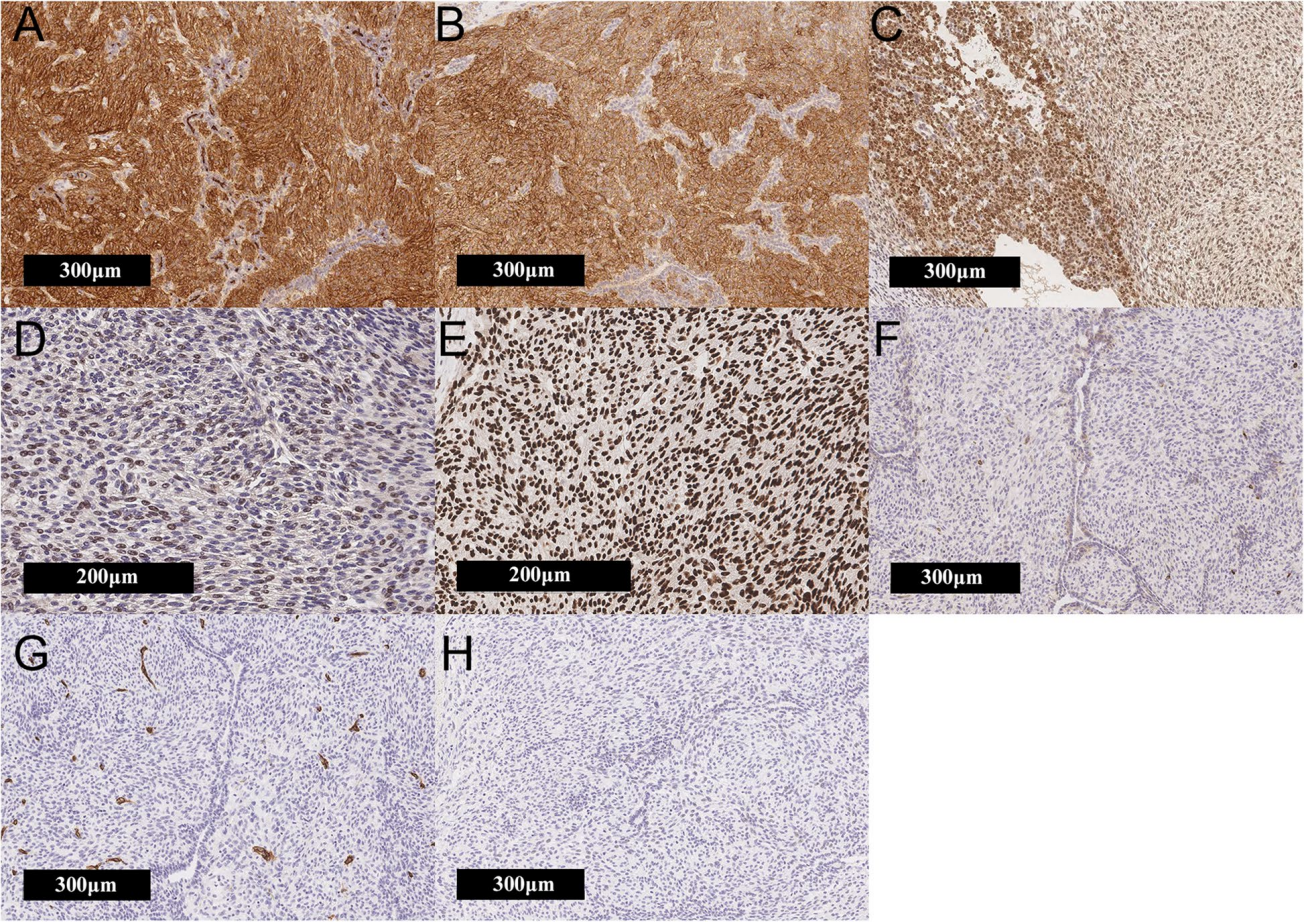
Immunohistochemical findings of the gastroblastoma. **A** Expression of CD10 by tumor cells (x100). **B** Expression of CD56 by tumor cells (x100). **C** Expression of GLI1 by tumor cells (x100). **D** Expression of PD-L1 by tumor cells (x200). **E** Expression of HDAC2 by tumor cells (x200). **F** Lack of KIT expression by tumor cells (x100). **G** Lack of CD34 expression by tumor cells (x100). **H** Lack of SS18-SSX expression by tumor cells (x100)

Grossly, the surgical specimen was revealed to be a nodular and well-circumscribed mass measuring 7 x 7 x 6 cm in the gastric antrum. The tumor grew as an expansive mass and involved the gastric wall structures. The cut surface showed a greyish-yellow and tan solid mass with cystic and hemorrhagic components (Fig. 2-A). Histologically, a biphasic morphology of epithelial and spindle cell components was observed. The epithelial components were slit-like glandular structures composed of low cuboidal epithelium with tubular or rosette-like differentiation with eosinophilic secretions. The spindle cell components consisted of short spindle-shaped oval cells infiltrating the smooth muscle. These cells had small round nucleoli and well-defined cell borders (Figs. 2-B, C, D). Mitotic activity was low in both components. Lymph-vascular invasion was not found. Immunohistochemical (IHC) analysis revealed that the spindle cell component was positive for vimentin, CD10 (Fig. 3-A), CD56 (Fig. 3-B), and glioma-associated oncogene homolog 1 (GLI1) (Fig. 3-C), focally positive for PD-L1 (Fig. 3-D), and histone deacetylase 2 (HDAC2) (Fig. 3-E). The epithelial component was positive for pan-cytokeratin (CK AE1/AE3), CAM5.2, and CK7, but negative for CK20 and epithelial membrane antigen (EMA). Both components were negative for KIT (Fig. 3-F), CD34 (Fig. 3-G), DOG1, smooth muscle actin (SMA), desmin, S100 protein, chromogranin A, synaptophysin, CDX2, and SS18-SSX (Fig. 3-H). Antibodies used for the IHC analysis are shown in Table 1, while results of the analysis are shown in Table 2.

**Table 1:** Summary of primary antibodies used in this report

**Primary antibody**	**Source**	**Clone**	**Dilution**	**Treatment**
KIT (CD117)	DAKO	Polyclonal	Ready to use	Heat retrieval (pH6.0)
CD56	DAKO	1B6	Ready to use	Heat retrieval (pH9.0)
CD10	DAKO	56C6	Ready to use	Heat retrieval (pH9.0)
PD-L1	DAKO	22C3	Ready to use	Heat retrieval (pH6.0)
Anti-HDAC2	abcam	HDAC2-62	1:1000	Heat retrieval (pH9.0)
SMA	DAKO	1A4	Ready to use	Heat retrieval (pH9.0)
CDX-2	DAKO	DAKO-CDX2	Ready to use	Heat retrieval (pH9.0)
CAM5.2	BectonDickinson	CAM5.2	1:20	Heat retrieval (pH9.0)
Cytokeratin	DAKO	AE1/AE3	Ready to use	Heat retrieval (pH9.0)
CD34	DAKO	NU-4A1	Ready to use	Heat retrieval (pH9.0)
Desmin	DAKO	D33	Ready to use	Heat retrieval (pH9.0)
Vimentin	DAKO	Vim 3B4	Ready to use	Heat retrieval (pH9.0)
SS18-SSX	Cell signaling Technology	SS18-SSX	1:500	Heat retrieval (pH6.0)
GLI1	Santa Cruz Biotechnology	C-1	1:500	Heat retrieval (pH6.0)
CK7	DAKO	OV-TL 12/30	Ready to use	Heat retrieval (pH9.0)
CK20	DAKO	Ks20.8	Ready to use	Heat retrieval (pH9.0)
EMA	DAKO	E29	Ready to use	Heat retrieval (pH9.0)
S100	DAKO	Polyclonal	Ready to use	Heat retrieval (pH6.0)
Chromogranin A	abcam	Polyclonal	1:100	Heat retrieval (pH9.0)
Synaptophysin	DAKO	DAK-SYNAP	Ready to use	Heat retrieval (pH9.0)
DOG1	Leica	K9	1:50	Heat retrieval (pH9.0)

**Table 2:** Summary of previously published gastroblastoma cases

**Case**	**Author (publication year)**	**Age (year)**	**Sex**	**Specimen type**	**Locus**	**Size (mm)**	**Chief complication**	**IHC positive**	**IHC negative**	**Fusion gene**	**Treatment**	**Metastases**	**Outcome**	**Follow-up (month)**
1-3	Miettinen et al. (2009) [1]	19, 27, 30	M, F, M	R	B, B, A	50, 60. 150	N/A, AP, anemia and fatigue	Vim (S), CD10 (S); CK (pan) (E); CK 18 (2/2, focally in one of the cases) (E); CK 7 (focally in E); DOG1 (focally in E, dot-like positivity in S)	CK20 (E); EMA (E); KIT (CD117) (E, S); CD34 (E, S); CD99 (E, S); SMA (E, S); Des (E, S); calretinin (E, S); p63 (E, S); S100 (E, S); CgA (E, S); Syn (E, S); CDX2 (E, S); and TTF-1 (E, S)	ND	SG, PG, antrectomy and radiation	No	ANED	42, 60, 168
4	Shin et al. (2010) [2]	9	M	R	A	90	AP and palpable mass.	CD10 (S); CD56; KIT (CD117) (E); CK (pan) (E); CK (LMW) (E); EMA (E); Vim (S)	Calretinin; CD34; CEA; CgA; CK (HMW); Des; inhibin; NSE; p63; SMA; Syn	ND	R	No	ANED	9
5	Wey et al. (2012) [3]	28	M	R	A	38	N/A	CD10; CD56; KIT (CD117) (E); CK (pan) (E); CK (LMW) (E); CK7 (focally in E); Vim (S)	CK20 (E); calretinin (E); CDX2 (E); Des (E); EMA (E); inhibin; p63 (E); S100 (E); SMA	ND	CHT followed by PG	LN, liver,	ANED	3
6	Femandes et al. (2014) [4]	19	F	R	A	105	AP	CD10 (S, focally in E); CD56; CK AE1/AE3 (E); CK CAM5.2 (E), Vim (S)	Calretinin; CD34; KIT (CD117); CgA; Des; DOG1; S100; SMA; Syn	ND	PG	No	ANED	20
7	Ma et al. (2014) [5]	12	M	R	A	45	Abdominal mass and blood in stool	CD10 (S); CD56 (S); CK AE1/AE3 (E); CK CAM5.2 (E); Vim (S)	ALK; CD34; KIT (CD117); CK5/6; Des; DOG1; PLAP; S100; SMA	ND	SG and gastroduodenostomy	No	ANED	8
8	Toumi et al. (2017) [10]	29	F	R	U	70	Epigastric pain	CD10 (focally); CD99	CK (pan); KIT (CD117); CgA; Syn	ND	R	LN	DEAD	6
9	Graham et al. (2017) [6]	28	M	R	A	38	N/A	CK (pan) (E), CD56 (E), NSE (E), low Ki-67	CgA, Syn, CEA, TTF-1, PLAP, CD30, AFP, HCG	*MALAT1– GLI1*	R NOS	LN, liver, peritoneum	N/A	N/A
10	Graham et al. (2017 [6])	27	M	R	A	ND	N/A	CK (pan) (E), SMA (patchy in S)	CgA, Syn, KIT (CD117), DOG1, Des, S100, melanA, SOX10, TLE-1, CD99, CK5/6	*MALAT1– GLI1*	R NOS	No	ANED	12
11	Graham et al. (2017) [6]	9	M	R	A	90	N/A	CK (pan) (E), CD10 (focally in S), KIT (CD117) (E, S), CD56 (E), Vim (S)	CgA, Syn, NSE, Des, SMA, calretinin, inhibin, CK34βE12, CD34	*MALAT1– GLI1*	R NOS	No	ANED	93
12	Graham et al. (2017) [6]	56	F	Needle biopsy	NS	40	N/A	OSCAR (patchy in E), low Ki-67, Vim (S)	CK34βE12, CKT7, CK20, CDX2, CgA, Syn, CD34, CD99, KIT (CD117), DOG1, calretinin, WT1, SMA, Des, EMA, MOC31, melanA, HMB45, pCEA	*MALAT1– GLI1*	Biopsy	Liver	N/A	N/A
13	Castri et al. (2019) [7]	79	M	R	A	90	Weight loss and dysphagia	bcl2; CD10 (S> E); CD56; CK (pan) (E> S); Vim (S)	aFP, bHCG, h-caldesmon, calponin, CD31, CD99, KIT (CD117), CgA, Des, DOG1, ERG, GFAP, HMB45, melanA, PLAP, S100, STAT6, Syn	*MALAT1– GLI1*	PG	Local relapse	ANED	52
14	Giovanni et al. (2019)	43	F	R	A	53	Intestinal bleeding	CD10 (focally in S), EMA (focally in E), CK (pan) (E), CAM 5.2 (E), CK 7 (focally in E), Vim (S), GLI1 (E, S)	SMA (E, S), Calretinin (E, S), CgA (E, S), NSE (E, S), CD34 (E, S), CD56 (E, S), CD99 (E, S), CDX2 (E, S), Des (E, S), DOG1 (E, S), ER (E, S), CK 5/6 (E), CK 20 (E, S), Inhibin (E, S), KIT (CD117) (E, S), p63 (E, S), Syn (E, S), S100 (E, S), TTF1 (E, S), TLE1 (E, S)	ND	PG	No	ANED	100
15	Koo et al. (2021) [8]	17	M	R	U	63	Intestinal bleeding	Vim (E, S), CD56 (E, S), CD10 (E, S), CK (pan) (E, S), Syn (E, S)	CD34 (E, S), KIT (CD117) (E, S), DOG1 (E, S), S100 (E, S), Des (E, S), CgA (E, S)	*EWSR1-CTBP1*	PG	No	ANED	23
16	Chen C et al. (2022) [11]	58	M	R	M	23	N/A	Vim (E, S), CD10 (E, S), bcl-2 (E, S), CD56 (E), CD100 (E), EMA (focal), Ki-67 (low)	KIT (CD117) (E, S), DOG1 (E, S), CD34 (E, S), SMA (E, S), Des (E, S), SOX10 (E, S), CD99 (E, S), CgA (E, S), syn (E, S), CAM5.2 (E, S), CK (pan) (E, S).	*PTCH-GLI2*	ESD	No	N/A	N/A
17	Present case / Sugimoto	28	F	R	A	70	AP	CK (pan) (E), CAM5.2 (E), CD10 (S), CD56 (S), Vim (S), GLI1 (S), CK 7 (E), PD-L1 (focally in S), HDAC2 (E, S)	CK20 (E, S), EMA (E, S), CDX2 (E, S), KIT (CD117) (E, S), CD34 (E, S), SMA (E, S), Des (E, S), S100 protein (E, S), CgA (E, S), Syn (E, S), DOG1 (E, S), SS18-SSX (E, S).	*MALAT1– GLI1*	PG	No	ANED	8

*Abbreviations:* *U *Gastric cardia and fundus, *B * Gastric body, *A * Gastric antrum, *AP * Abdominal pain, *IHC * Immunohistochemical stain, *ANED * Alive no evidence of disease, *CK * Cytokeratin, *Des * Desmin, *Vim * Vimentin, *SMA * Smooth muscle actin, *TTF-1 * Thyroid transcription factor 1, *CgA * Chromogranin A, *Syn * Synaptophysin, *S * Spindle cell component, *E * Epithelial cell component, *N/A * Not available, *ND * Not done, *SG * Subtotal gastrectomy, *PG * Partial gastrectomy, *R * Resection, *LN * Lymph node

**Fig. 4:**
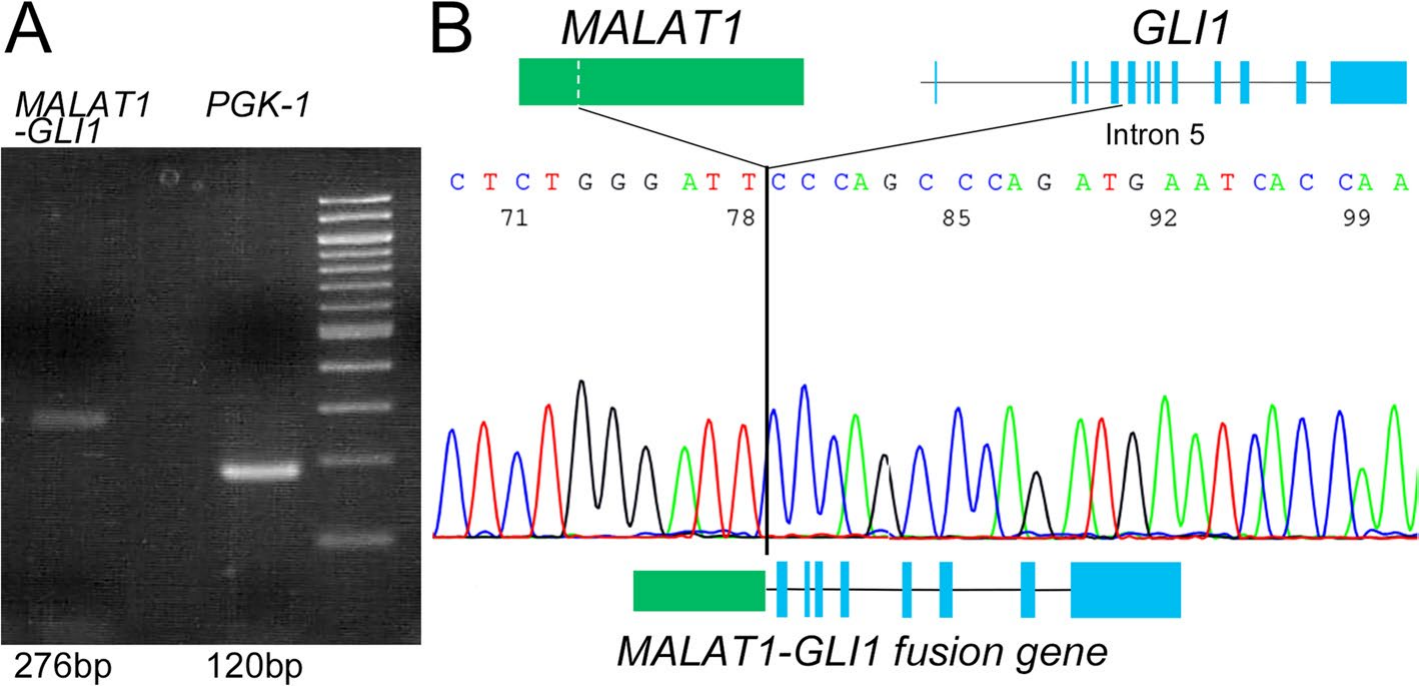
A *MALAT1–GLI1* fusion gene detected by molecular analysis. **A** Confirmation of the presence of a *MALAT1–GLI1* fusion transcript by RT-PCR analysis. **B **Sequencing of the cDNA-confirmed fusion of *MALAT1* and *GLI1*

We performed reverse transcriptase-polymerase chain reaction (RT-PCR) analysis [6], which revealed that the tumor harbored the *MALAT1–GLI1* fusion gene (Fig. 4-A, B). In addition, we developed a customized next-generation sequencing (NGS) gene panel for use in this case. The panel consisted of 28 genes (*APC, TP53, CDKN2A, MET, ATM, MLH-1, PMS2, HRAS, AXIN2, BAX, DCC, MSH2, POLE, RNF43, PTEN, BRAF, EPCAM, MSH6, BUB1B, RhoA, KRAS, NRAS, SMAD4, CDK4, PIK3CA, STK11, TGFBR2,* and *EGFR*) for exploring the genetic causes of colorectal cancer. This panel was employed for gastroblastoma in the present case to detect gene mutations. However, positive pathogenic / likely pathogenic variants were not detected with this NGS panel.

## Discussion and conclusions

To our knowledge, 16 cases of gastroblastoma have been reported in the medical literature. Table 2 summarizes the clinicopathological features of these cases [1–11], as well as the clinicopathological findings associated with the present case. Nuclear PD-L1 and HDAC2 expression was observed in the spindle cell component by IHC analysis. PD-L1 is transported from the cell membrane into the nucleus and activates other checkpoint inhibition-related genes. PD-L1 transport into the nucleus was shown to be regulated by HDAC2 [12]. In the present case, PD-L1 and HDAC2 were co-expressed in tumoral nuclei. We suggest that PD-L1 migrated into the nucleus via intranuclear HDAC2 activation. As a result, we speculate that HDAC inhibitors may offer a promising treatment option for gastroblastoma. Surgical resection is the standard treatment for gastroblastoma; however, in a small number of cases, chemotherapy or radiotherapy was used [1, 10]. The postoperative course of the disease is generally favorable. However, a few cases of postoperative local recurrence, distant metastases and death have been reported [3, 6, 7, 10]. Therefore, it is valuable to mention feasible treatment options; in this case, we showed nuclear migration of PD-L1 and overexpression of HDAC2, suggesting that HDAC2 inhibitors may be helpful. However, as this was a single case report, further studies are needed to confirm this result.

**Fig. 5:**
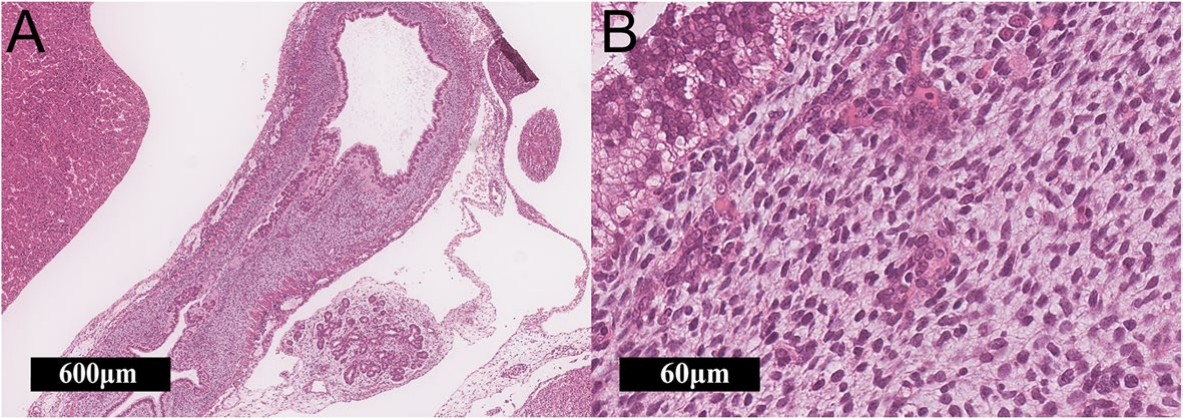
Histology of the fetal gastrointestinal tract (at 10 weeks). **A**, **B** Mesenchyme of the gastrointestinal tract, which resembles the spindle component of the gastroblastoma (**A**: x100, **B**: x200)

The tumorigenesis of gastroblastoma is not completely understood. Although Toumi et al. reported that gastroblastoma is believed to develop from a totipotent stem cell, the relationship between gastroblastoma and stem cells is still unclear [10]. Histologically, the embryonic gastrointestinal mesenchyme is morphologically analogous to gastroblastoma (Fig. 5-A, B). In the development and differentiation of the gastrointestinal system, the epithelium and mesenchyme exhibit crosstalk via molecular signaling pathways, such as FGF, TGF-b, Wnt, Hippo, Notch and Hedgehog (Hh). In particular, the Hh signaling pathway is the common pathway for embryo and gastroblastoma development [13]. Gastroblastoma activates GLI1 transcription by the *MALAT1-GLI1* fusion gene. We suggest that the morphological similarities between the tumor and the embryonic gastrointestinal mesenchyme might be due to the effect of GLI1 expression via the Hh pathway.

In conclusion, we report the following new findings associated with a case of gastroblastoma: (i) gastric tumors mimic the gastrointestinal mesenchyme in the embryonic period and (ii) nuclear expression of PD-L1 and HDAC2 were observed. We speculate that HDAC inhibitors may offer a promising treatment option for gastroblastoma.
